# What is Resilience? A Case Report of a Fully-Functional Man with Corpus Callosum Agenesis

**DOI:** 10.1192/j.eurpsy.2022.2285

**Published:** 2022-09-01

**Authors:** E. Ferry-Bolder, E. Mak, K. Ersche

**Affiliations:** University of Cambridge, Department Of Psychiatry, Cambridge, United Kingdom

**Keywords:** Neuroimaging, Corpus callosum agenesis, resilience, Brain abnormailty

## Abstract

**Introduction:**

Resilience has attracted much attention, not least since the pandemic. It is characterised by a person’s ability to bounce back from adversity. Although there is no exact definition of what adversity means, it assumes that the individual is aware of the event. Here, we would like to challenge this preconception by putting forward a case of an individual who shows remarkable resilience without being aware of his disadvantages.

**Objectives:**

We present a case of a fully-functioning middle-aged man with corpus callosum agenesis of which he has been unaware. We sought to demonstrate that personality traits which have been associated with resilience may not necessarily be characteristic of a resilient individual.

**Methods:**

T.C. is a 44-year-old individual who enrolled as a healthy participant in a research study at the University of Cambridge, which involved cognitive and personality assessments and a structural brain scan.

**Results:**

T.C.’s psychological profile portrayed a well-balanced man who had attained a high level of education, stable employment, a healthy personal life and good community integration. T.C.’s cognitive performance fell well within normal ranges, but was superior in terms of self-control, as measured by the stop-signal task. To our surprise, he scored below-average on questionnaires of resilience and sense of coherence beliefs and reported subclinical tendencies of obsessive-compulsive behaviours.

**Conclusions:**

Resilience does not require awareness of adversity. Our case report shows that resilience may present itself fairly normally and may go unrecognised in daily life. Hardship should not be limited to traumatic events but also include brain abnormality.

**Disclosure:**

No significant relationships.

